# The Longevity of Bridge-Work

**Published:** 1900-10-15

**Authors:** H. Baldwin

**Affiliations:** M.R.C.S.


### THE LONGEVITY OF BRIDGE-WORK.

BY H. BALDWIN, M.R.C.S., L.D.S.

Read at the meeting of the Metropolitan Branch of the British Dental Association, April 25, 1900.

It may be well to preface my remarks by the observation that my practical acquaintance with a certain class of bridges is derived almost entirely from other people's work which has been generally a failure. This may possibly have given a somewhat biased point of view, but the unfavorable results I have seen are only the confirmation of certain beliefs held which are based upon theoretical considerations. This being so, I feel inclined to pass in review considerations which refer to the advantages and disadvantages (virtues and vices) of plate-work and bridge-work respectively, and then to consider if by any means the virtues which ordinarily belong to the two classes of work separately can be combined in one of them. Of course I leave out of the question all those cases where bridge-work is entirely impossible, and concern myself only with those which enthusiastic bridge builders would consider typical cases for their work.

To begin with plate-work.

The advantages of plate work are just the counterpart of the disadvantages of bridge-work.

The disadvantages of bridge-work are:

1. That a larger number of artificial teeth are fixed to a smaller number of roots than nature intended.

2. That the roots to which the bridge is fixed are immovably united together, which is the reverse of what nature intended.

3. That the very useful support which is offered by the bone of the alveolar process and by the gum is neglected.

4. That the articulation of bridges for masticating pur poses is never so good as that of a well-made plate.

5. That bridges in the making often present a great temptation to mutilate sound teeth.

6. They are difficult to alter or repair.

7. That the temptation exists for a patient to go on wearing a bridge for long after it has become useless for mastication, owing to loosening of the roots.

On the other side of the picture are the advantages of bridge-work, which may be summed up under three heads:

1. That no large portion of the gum is covered by the work.

2. That the work is not to be removed at night.

3. That the natural teeth in the vicinity are not so likely to be damaged by caries.

With regard to the disadvantages of bridge-work, we have seen that a larger number of teeth are fixed to a smaller number of roots than nature intended, and the very efficient support of the gum and alveolar process is discarded, also that the roots or teeth which serve as the foundations of bridge-work are often immovably fixed together, whereas nature arranged that they should have a slight lateral play in mastication. What then theoretically would one expect to happen to a large bridge which is fully opposed to the force of mastication? One would expect, first, that the roots serving as foundations would be in time loosened by the abnormal strain, and second, that the bridge would try hard to crack loose from some of its attachments. Both these things are exactly what happens. The question arises how far the normal relation between the number of teeth and number of roots can be interfered with, with impunity. Personally, I require two roots for every three artificial teeth supported by them.

When there are a large number of roots available, a bridge is possible, but it is in just this class of case that a plate loses its characteristic disadvantages and combines all the virtues of both bridge and plate. This combination of virtues is to be attained by first crowning all the roots and broken-down teeth, then by constructing a very narrow plate resting on the alveolar ridge only, and clasping the crowns by way of attachment. The crowns are in these cases to be specially constructed with parallel sides to allow the plates to slip on and off easily, and yet to fit closely. This denture may be worn at night with as much propriety as a bridge, because its fixations are all upon crowned teeth not susceptible to caries. This method is much to be preferred to making a large bridge, as it embodies all the advantages of a bridge, with the additional supreme advantage of utilizing the support of the alveolar ridge, which is the best possible support for artificial teeth. The making of large bridges would then appear at all times to be mistaken practice. Small bridges supported by good roots in the proportion of two roots to three artificial teeth I am in favor of, but I limit my bridge-building to such small cases. In practice, I find that small very narrow plates, made with special care as to bands, and made from a plaster impression, are very satisfactory. The more I see of fixing various roots tightly together, the less I like it. If I have two roots to crown, one good and one bad, I prefer to crown them separately rather than fix them together. If fixed together the attachment to each root must be so strong as to be able to support the double force of mastication acting on the two teeth. This is obviously so, because neither root is held rigidly in its socket. There is thus a distinct loss of strength by fixing them together. Rather than fix them together in such a case, I would fix the two crowns on to the one good root and discard the other, or simply let the second crown rest upon the worse root, this one being previously filled to prevent its speedy loss by decay.

To sum up, I would say:

1. Make no large bridges, but crown all serviceable roots and broken-down teeth, and then construct a narrow plate—very narrow, if you like—merely the width of the alveolar ridge—and attach it by clasps to the crowns.

2. Reserve bridge-making for small cases, chiefly where one tooth only is entirely absent, or where the bite is feeble.

3. Never fix two or more teeth or roots together if you can possibly keep them apart.

4. Always utilize the solid support of the alveolar process as a basis for artificial teeth when the pressure and force of mastication are at all considerable, excepting the selected small cases refered to.

5. Wherever any bridging is admitted, make the attachments of the bridge to the roots immensely strong or else intentionally movable.—*Journal of British Dental Association.*

